# Cecropin-like antimicrobial peptide protects mice from lethal *E*.*coli* infection

**DOI:** 10.1371/journal.pone.0220344

**Published:** 2019-07-25

**Authors:** Anishma Shrestha, Deepesh Duwadi, James Jukosky, Steven N. Fiering

**Affiliations:** 1 Colby-Sawyer College, New London, NH, United States of America; 2 Department of Microbiology and Immunology, Geisel School of Medicine at Dartmouth, Lebanon, NH, United States of America; Università degli studi di Napoli Federico II, ITALY

## Abstract

Resistance of pathogenic bacteria to standard antibiotics is an issue of great concern, and new treatments for bacterial infections are needed. Antimicrobial peptides (AMPs) are small, cationic, and amphipathic molecules expressed by metazoans that kill pathogens. They are a key part of the innate immune system in both vertebrates and invertebrates. Due to their low toxicity and broad antimicrobial activities, there has been increasing attention to their therapeutic usage. Our previous research demonstrated that four peptides—DAN1, DAN2, HOLO1 and LOUDEF1—derived from recently sequenced arthropod genomes exhibited potent antimicrobial effects *in-vitro*. In this study, we show that DAN2 protected 100% of mice when it was administered at a concentration of 20 mg/kg thirty minutes after the inoculation of a lethal dose of *E*. *coli* intraperitoneally. Lower concentrations of DAN2—10mg/kg and 5mg/kg protected more than 2/3s of the mice. All three dose levels reduced bacterial loads in blood and peritoneal fluid by 10-fold or more when counted six hours after bacterial challenge. We determined that DAN2 acts by compromising the integrity of the *E*. *coli* membrane. This study supports the potential of DAN2 peptide as a therapeutic agent for treating antibiotic resistant Gram-negative bacterial infections.

## Introduction

Prior to the discovery of antibiotics, the majority of deaths in all age groups were associated with infection by pathogenic bacteria. Over the past 80 years, conventional antibiotics have been routinely used to treat bacterial infections, and this has markedly reduced morbidity and mortality from bacterial infections. The widespread feeding of antibiotics to farm animals for non-therapeutic purposes and an excessive use of antibiotics to treat people who often have no bacterial infection has selected for bacterial strains that resist multiple antibiotics. The threats from multidrug resistant bacteria such as Methicillin-resistant Staphylococcus aureus (MRSA), extended-spectrum beta-lactamase (ESBLs) multi-drug resistant tuberculosis (MDR), and all-antibiotic resistant superbug gonorrhea are increasing at an alarming rate worldwide [1,2]. According to the WHO report, there were 490,000 cases of multi-drug resistant TB globally in 2016 [2]. Increasing the available treatments for antibiotic resistant bacteria is necessary to prevent mortality and morbidity due to these infectious agents. In this context, the potential of antimicrobial peptides (AMPs) as promising candidates for novel antimicrobial agents deserves attention.

The biological world abounds in antimicrobial peptides since, for many organisms, they are the primary antimicrobial defense against bacteria, fungi and viruses. Over millions of years, natural selection of organisms that survive infections has driven the diversity of antimicrobial peptides, and metazoans have all evolved unique cohorts of antimicrobial peptides. The immense number, structural diversity, and multiple modes of action of these AMPs confers advantages when dealing with antibiotic resistance [3,4]. These peptides are usually 12–100 amino acids long, positively charged and amphipathic [5]. The positive charge is a consistent feature of these peptides and likely supports electrostatic interaction with negatively charged bacterial membranes. These highly positively charged amino acids rich in arginine and lysine have been shown to be important in AMP-mediated killing of various food borne pathogens [6]. More than 2,500 AMPs have been identified in various organisms [7]. Several peptides have been used clinically or are in clinical trials to treat bacterial infection, chronic wound healing, cystic fibrosis and other pathologies characterized by difficult-to-treat infections. Polymyxins, lipopeptides discovered in 1947, and colistin are cytotoxic peptides that are clinically used as last resort drugs for patients with multi-drug resistant bacterial infection [8]. The LTX series of synthetic antimicrobial peptides have promising antibacterial activity against *Staphylococcus aureus* infections [9]. Daptomycin, a lipopeptide, is active against Gram-positive bacteria only [10]. P-113, a histidine rich antimicrobial peptide that is originally derived from human saliva, has potent activity against fungal infections in HIV patients with oral candidiasis [11]. These peptides all display rapid action of killing and low minimum inhibitory concentrations (MIC) [8].

Every organism has a defense system against pathogenic infections [12]. The highly sophisticated vertebrate defense system utilizes both an innate and an adaptive immune system whereas invertebrates, for the most part, lack the latter [13]. AMPs are integral components of immunity in multicellular animals and play a major role in protecting these organisms from pathogens. Individual AMPs vary in efficacy against different classes of pathogens, but AMPs as a group have activity against all classes of single-cell pathogenic microorganisms, including Gram-positive and Gram-negative bacteria, protozoa, yeast, fungi, and viruses. Antimicrobial peptides are grouped structurally and by sequence and organism source as cecropins, insect-defensins, glycine-rich proteins, proline-rich proteins.

A variety of cecropins and insect defensins have been studied and reported in the literature [14,15]. Cecropins are a family of cationic antimicrobial peptides of 31–39 residues with a broad spectrum of activity against Gram-negative and Gram-positive bacteria, as well as fungi. They were first isolated from the hemolymph of the giant silk moth, *Hyalophora cecropia* [16]. They lack cysteine, so they cannot form disulfide bridges [17]. Another large group of AMPs, defensins are 28–42 (~4 kDa) cationic AMPs with six conserved cysteine residues that can form three disulfide bridges [15,18]. They typically affect Gram-positive bacteria [15,18].

In our previous research, we have identified putative insect AMPs by searching newly sequenced arthropod genomes using known AMPs as homology templates [19]. We screened six cecropin and defensin derived peptides-DAN1, DAN2, HOLO1, LOUDEF1, INVICT1, and IXI for antimicrobial activity *in vitro* against several microbes including Gram-positive bacteria, Gram-negative bacteria and a single fungus. The results from radial diffusion assays and broth microdilution assays demonstrated potent antimicrobial activities of four peptides—DAN1, DAN2, LOUDEF1, and HOLO1. Cecropin family members, DAN1 and DAN2, were most effective against Gram-negative bacteria including *E*. *coli* and *P*. *aeruginosa*. In addition, these peptides were not toxic to mammalian cells even at concentrations ten and twenty times higher than the minimum inhibitory concentration (MIC) to inhibit the growth of bacteria as demonstrated by minimal hemolysis of sheep erythrocytes [1].

*In vitro* studies for efficacy against Gram-negative bacteria and lack of toxicity against erythrocytes encouraged us to perform more definitive *in vivo* preclinical studies to determine whether DAN2 could protect against lethal infection without overt toxicity. In this paper, we present the results of DAN2 treatment of acute lethal infection with *E*. *coli* and its mechanism of microbicidal activity.

## Materials and methods

### 2.1. Synthesis and application of peptide

DAN2 was identified in the inferred translation of genomic sequence data from the monarch butterfly (*Danaus plexippus*). The peptide was commercially synthesized by GenScript (Piscataway, NJ), which utilized solid phase peptide synthesis, and purified the peptide using HPLC to >85% purity. We dissolved the peptide in 0.01% acetic acid solution and stored it at -70°C in working stock solutions (5 mg/mL) for antimicrobial assays. An aliquot of stock solution was diluted in sterile PBS solution before administering it into mice.

### 2.2. Bacterial strains

*E*.*coli* strain ATCC 25922 (Serotype 06, Biotype 1) was purchased from American Type Culture Collection. This is a standard laboratory and non-multi drug resistant strain that does not express a toxin.

### 2.3. Mouse strains

Female C57BL/6 mice (6–8 weeks of age, approximately 20 g) were obtained from Jackson Laboratory. Animal studies were specifically approved by the IACUC of Dartmouth (Protocol number 00002014). They were kept in a temperature-controlled room under a 12 h light 12 h dark cycle with free access to commercial solid food and water. The mice were anesthetized using isoflurane prior to drawing blood and euthanized by an approved method while under anesthesia.

### 2.4. Preparation of bacteria

Bacteria were cultured in Mueller Hinton Broth (MH broth) with aeration at 37°C for 12 hours to obtain a stationary growth phase. On the day of infection, a fresh culture was made by inoculating bacteria into MH broth to make a final 100-fold dilution. The number of viable bacteria in the fresh culture was estimated based on the optical density at 600 nm. After doing manual colony counts, we calculated that an O.D._600_ of 0.4 contains approximately 2.0 × 10^8^ CFU/ml *E*. *coli* (ATCC 25922). The bacteria were washed with sterile PBS two times (8700 rpm for 5 mins) and re-suspended in PBS before administering to mice.

### 2.5. Mouse infection model

Mice were inoculated intraperitoneally (i.p.) with 2.2*10^7^CFU, (130 μl) of *E*. *coli* ATCC 25922. 30 minutes after bacterial challenges, the control mice received PBS by i.p. injection whereas the treatment mice received peptide by i.p. injection. Mice were monitored at least every hour for the first 24 hours. We observed that the infected mice started showing symptoms of illness such as hunched posture and ruffled fur 6–8 hours after bacterial challenge. Some of these mice appearing ill would recover and the endpoint criteria used was lack of responsiveness, at which point the animals were euthanized. Animals that had symptoms of illness were monitored every 30 minutes until they either appeared normal or were euthanized. Mice that survived for the first 24 hours were monitored daily for 5 days after infection. None of the mice that survived the 24 hours after bacterial challenge had observable symptoms of illness during subsequent days.

### 2.6. Bacterial counts in blood and peritoneal lavage

Bacterial counts were determined from blood and peritoneal fluid after 6 hours of bacterial challenge using a protocol described previously [20]. After injecting mice with 2.2 × 10^7^ CFU of *E*. *coli* and different doses of DAN2, the mice were anesthetized using isoflurane prior to drawing blood. The blood was collected retro-orbitally using a heparinized capillary and placed in a heparinized Eppendorf. Mice were then euthanized by cervical dislocation while still under anesthesia. 3 mL of PBS was then injected into mice intraperitoneally, and the abdomen was gently massaged. Approximately 1 ml of fluid was drawn using a syringe and collected in a tube. Blood and peritoneal fluids were then diluted to an appropriate dilution, from which 100 μl was plated on MH agar plate. The plates were incubated overnight at 37° C and colonies were counted manually after 12–18 hours of incubation. A total of 20 mice were used to determine the bacterial loads in blood and peritoneal fluid in mice treated with different concentration of DAN2.

### 2.7. Flow cytometric analysis

The integrity of the bacterial membrane after the treatment with DAN2 was determined via staining with Propidium Iodide (PI)[21,22]. If there are holes in the membrane, PI enters the bacteria, binds to DNA and fluoresces. The bacterial strain was grown to the exponential phase (O.D._600_ = 0.4) and re-suspended in PBS to a final concentration of 10^6^ CFU/ml after washing twice with PBS. Bacteria were mixed with DAN2 at a concentration of 24 μg/ml (twice the MIC) and were incubated for 30 min at 37° C. The bacteria were stained with PI solution (1 mg/ml) for 10 minutes at room temperature in the dark. Flow cytometric measurements were performed and the data were evaluated using Flojo software. Heat killed bacteria was used as a positive control. The bacterial solution was incubated in flow cytometry tube at 85° C water bath for five minutes before adding PI.

## Results

### 3.1. DAN2 protects mice from lethal bacterial challenge

The antimicrobial effect of DAN2, confirmed by several *in-vitro* tests such as the radial diffusion test and a broth micro-dilution assay, was assessed for the ability to protect mice from infection using an acute mouse model of infection. Bacterial concentration of 2. 2 × 10^7^ CFU was determined to be a lethal concentration in all mice (LC_100_). We challenged mice with this concentration of bacteria by i.p injection and treated i.p. with different concentrations of DAN2 30 minutes after infection. Infected untreated mice started showing symptoms 6–8 hours after bacterial challenge and reached the endpoint within 12 hours.

A preliminary experiment involved six mice in a treatment group that received 20 mg/kg of peptide and six mice in a control group that received PBS only after 30 minutes of bacterial challenge. All the mice treated with 20 mg/kg survived for five days, while all the control mice reached the endpoint within 12 hours (S1 Fig). In order to determine the least effective concentration that could protect all the mice from lethality, we performed another set of experiments with four different concentrations of DAN2 (0 mg/kg, 5 mg/kg, 10 mg/kg, and 20 mg/kg).

**Fig 1:**
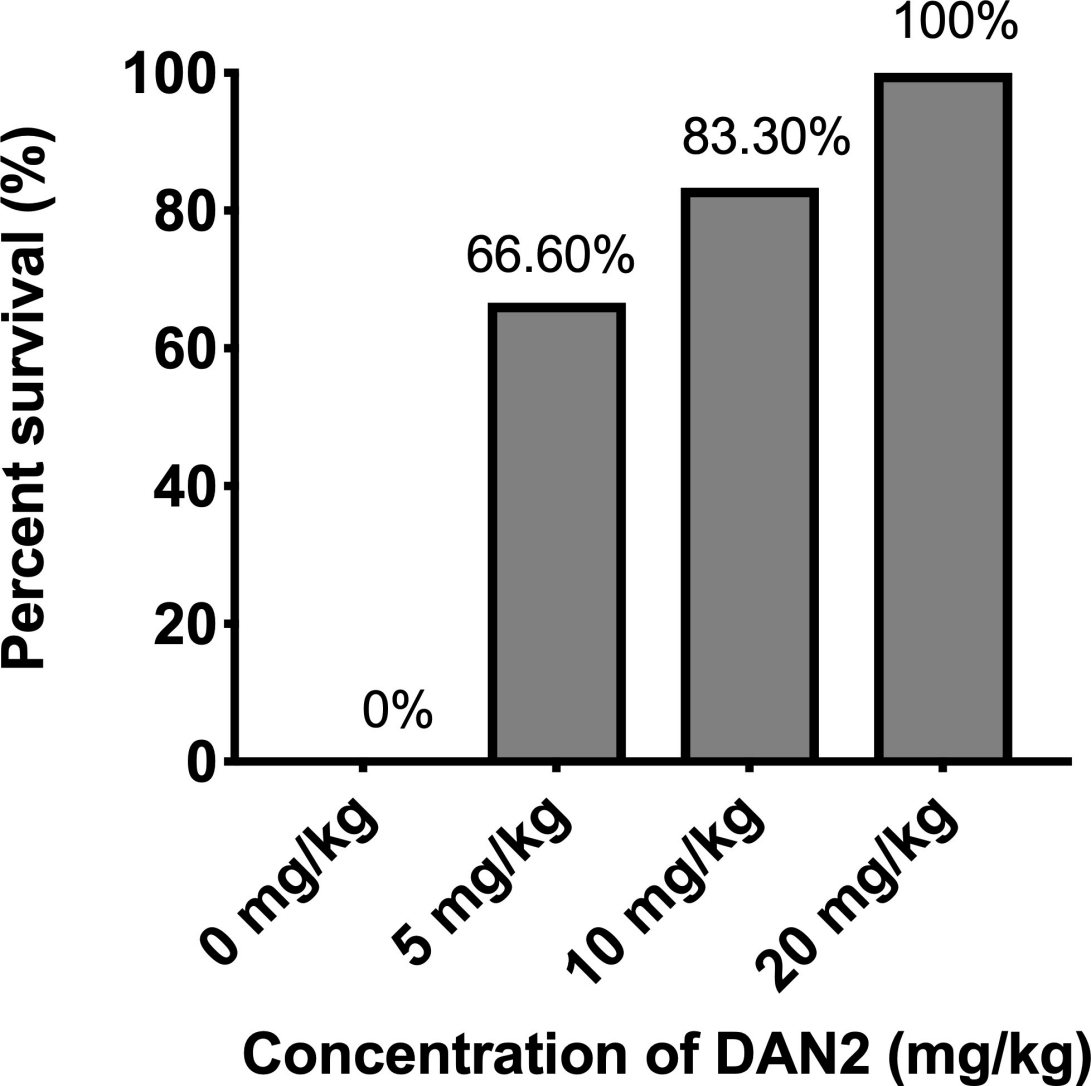
The infected mice exhibited dose-dependent response to survival. All the mice of four groups were injected intraperitoneally (i. p.) with a lethal dose, 2.2 × 10^7^ CFU of *E*. *coli* and treated with different concentration of DAN2 after 30 minutes of bacterial challenge. The control group only received bacterial suspension and PBS. All the treated mice were monitored for five days. There were 6 mice/group.

As shown in the Fig 1, when the mice were treated with 5 mg/kg and 10 mg/kg of DAN2, we observed survival rates of 67% and 83% respectively. Out of six mice that received 5 mg/kg, two mice reached the endpoint in 20 hours, while one mouse that received 10 mg/kg reached the endpoint at around 22 hours (S2 Fig). This indicates that 5 mg/kg and 10 mg/kg of DAN2 prolonged the survival of those mice. However, all the infected mice that were treated with 20 mg/kg of DAN2 survived, demonstrating that the effective dose 100 (EC_100_) of DAN2 is 20mg/kg.

### 3.2. DAN2 reduces bacterial counts in blood and peritoneal fluid after 6 hours of challenge

In order to document the impact of DAN2 on bacterial counts *in vivo*, we assayed bacterial loads in blood and peritoneal fluid in mice that received varying doses of peptide. This approach assesses the microbicidal or microbiostatic activity of DAN2. 4 mice were used in each treatment and were infected with 2. 2 × 10^7^ CFU. All mice in a group received a given concentration of DAN2 (0 mg/kg, 5 mg/kg, 10 mg/kg and 20 mg/kg) after 30 minutes of bacterial infection. Peritoneal fluid and blood were collected after 6 hours of bacteria challenge and the blood or peritoneal wash was plated in dilutions to provide bacterial counts.

**Fig 2:**
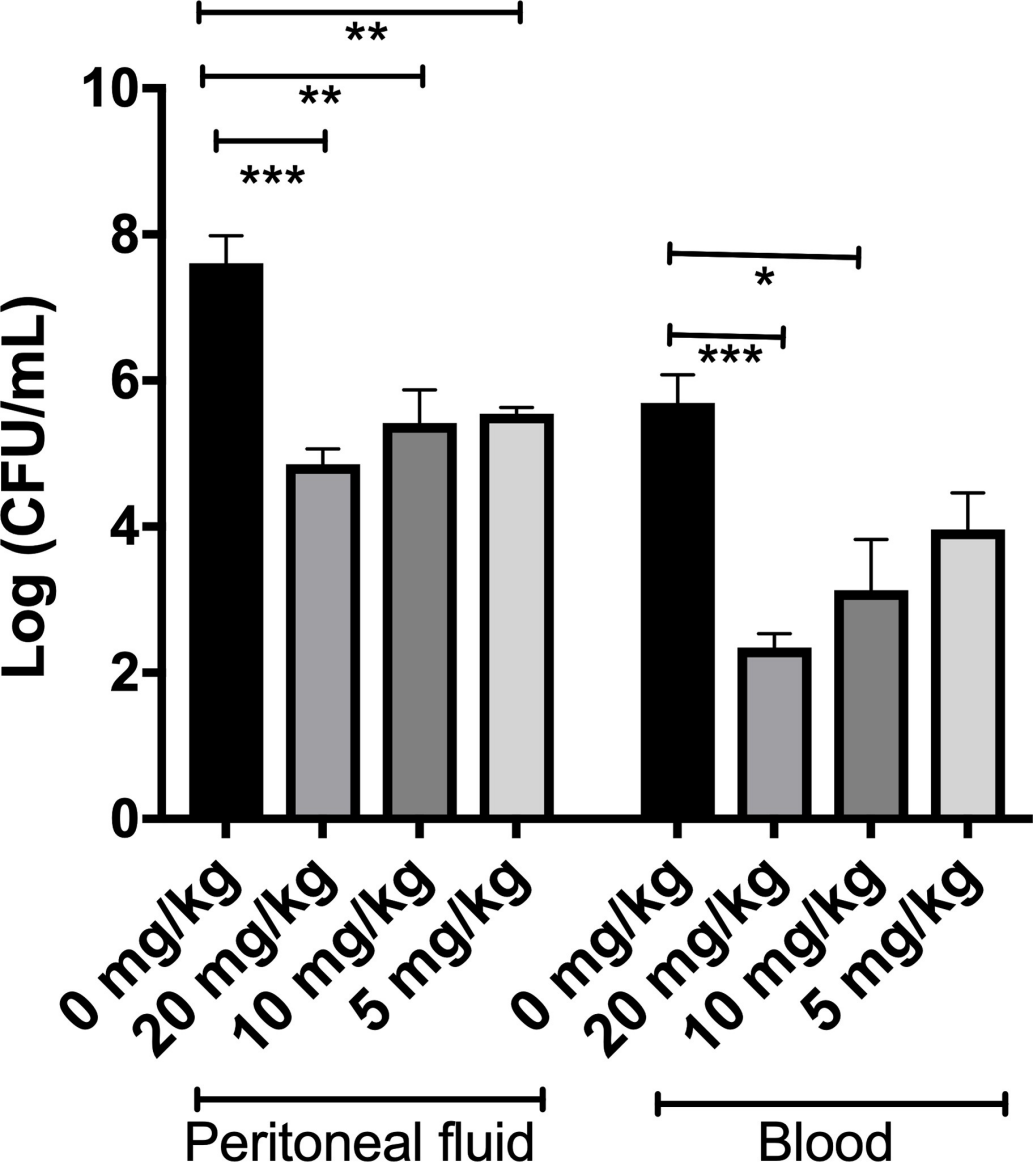
DAN2 treated mice have reduced bacterial loads both in peritoneal fluid and blood. Four mice were infected and treated with 0 mg/kg, 20 mg/kg, 10 mg/kg and 5 mg/kg of DAN2 individually and bacterial count was determined after 6 hours of infections. The error bars represent the standard deviation of the mean. The number of asterisks was used to denote the extent of statistical significance amongst groups (* denotes *p* < 0.05, ** denotes p < 0.005, *** denotes p < 0.0005).

As shown in Fig 2, bacterial loads in peritoneal fluid of the treated mice were significantly lower than that of the control mice and similar to each other regardless of the DAN2 dose. There was an approximately 150-fold decrease in bacteria in the treated mice compared to control mice after 6 hours of infection. One-way ANOVA analysis was performed to compare the effect of DAN2 dose on bacterial loads. A significant difference was observed in the bacterial loads between the experimental treatment groups (F _3, 11_ = 17. 8, *p* = 0.0002). Post-hoc comparisons using Tukey HSD tests (α = 0.05) indicated that there was a significant reduction in bacterial growth in mice treated with 20 mg/kg when compared with the control mice (*p* < 0.0005). Similarly, the difference between 10 mg/kg and control or 5 mg/kg and control was also statistically significant (*p* < 0.005).

Similarly, the bacterial load in blood was found to have decreased approximately 100-fold in mice treated with 20 mg/kg and 10-fold in mice treated with 10 mg/kg in comparison to the control mice. There was no significant decrease in bacteria in the mice treated with 5 mg/kg compared to PBS-treated mice. An analysis of variance (ANOVA) yielded significant difference in bacterial loads between the control and each treatment group (F _3, 12_ = 12.57, p = 0.0005). Post-hoc comparisons using Tukey HSD tests (α = 0.05) indicated that there was a significant reduction in bacterial growth in blood in mice treated with 20 mg/kg as compared to the control mice (p < 0.0005). The difference between 10 mg/kg and control was also statistically significant (p < 0.05). However, a statistical significance was not observed between control and 5 mg/kg (p > 0.05).

### 3.3. DAN2 permeabilizes *E*.*coli* membrane

**Fig 3:**
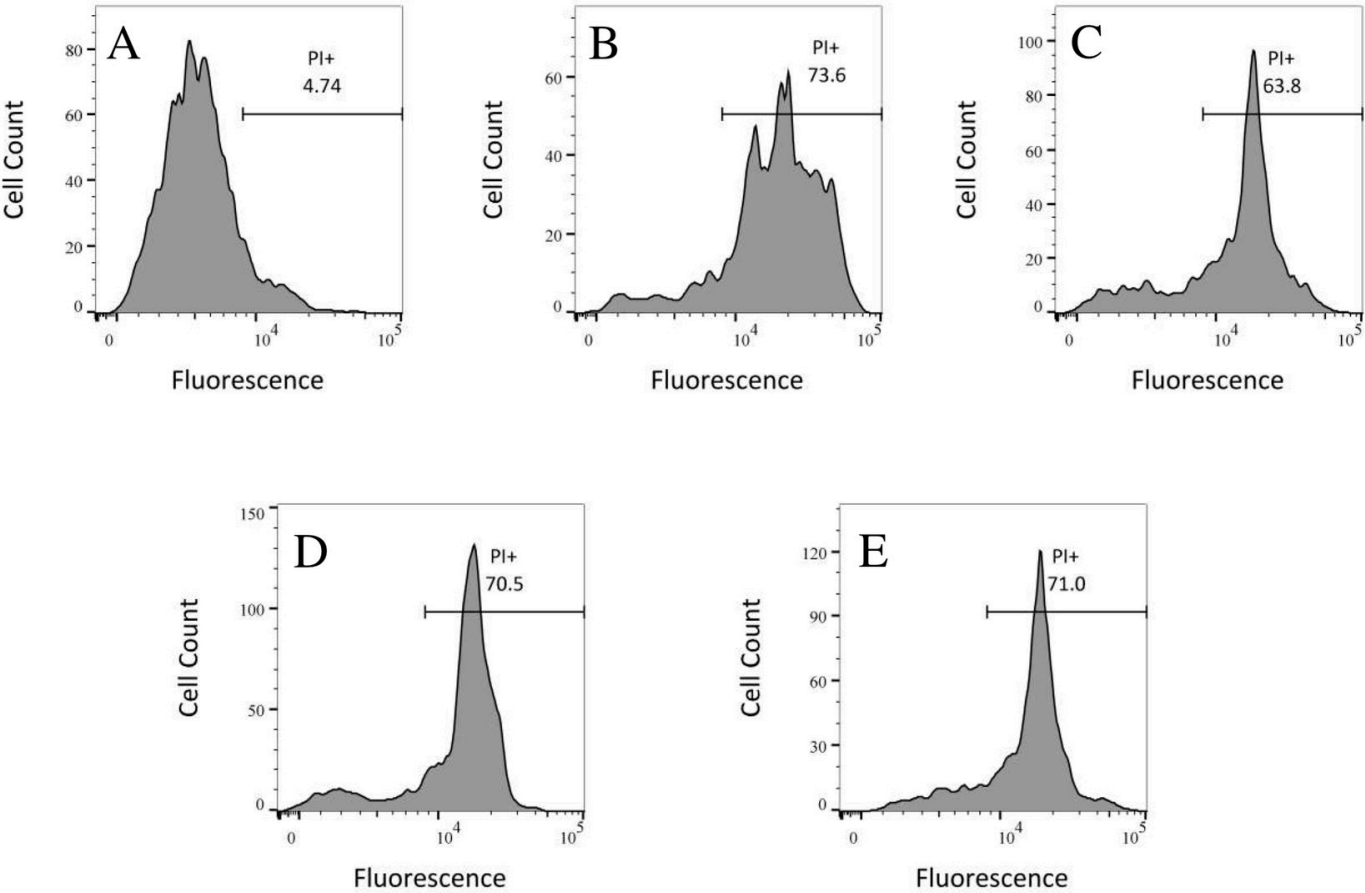
DAN2 disrupts the integrity of *E*.*coli* cell membrane. The DNA binding dye propidium iodide (PI) was used to evaluate cell membrane permeability of *E*. *coli* ATCC 25922 via flow cytometry. 2.0 × 10^6^ CFU/ml was incubated with varying concentrations of peptide for an hour and PI added subsequently. Flow cytometry was performed using a FACScan instrument. (A) Bacteria; (B) Heat treated (positive control); (C) DAN2 (48 μg/ml); (D) DAN2 (36 μg/ml); (E) DAN2 (24 μg/ml). Bacterial cells treated with either peptide or heat shocked have increased cellular fluorescence intensity of PI.

One identified mechanism of antimicrobial action of AMPs is forming pores in cytoplasmic membranes which if not repaired quickly is lethal. We evaluated the integrity of the cell membrane of *E*. *coli* using propidium iodide (PI), a DNA interacting dye that intercalates into DNA of permeabilized membrane and fluoresces brightly. The fluorescence of PI in a bacterial culture demonstrates that the membrane integrity is compromised. Fig 3 shows the fluorescence intensity of bacteria under different conditions. The bacterial cells treated with any of the tested concentrations of peptide or heated to 85° C have large fractions of the population that have admitted PI and fluoresce intensely. The area under the peak of higher fluorescence quantitates the cell population. Most of the heat shocked bacterial cells (74%, Fig 3B) were fluorescently labeled compared to the cells treated with peptide. The percentage of fluorescently labeled cells were 64%, 70% and 71% for the cells treated with 48 μg/ml, 36 μg/ml and 24 μg/ml of DAN2 respectively. Surprisingly, the intensity of fluorescing bacteria did not increase in proportion to the increasing concentration of DAN2, which indicates that many cells had compromised membranes at all of these treatment concentrations (Fig 3C, 3D and 3E). None of the samples, either heat shocked or peptide treated, showed any colony growth when 100 μl of the samples were plated for colony count showing that virtually all the bacteria were unable to divide, although not all fluoresced at the time of the assay.

## Discussion

In the presence of the global threat from antibiotic resistance, antimicrobial peptides are promising anti-infective agents. AMPs are particularly attractive because microbes are less likely to develop resistance [23,24]. AMPs primarily target bacterial cell membranes, and it is challenging for bacteria to preserve cell membrane structure and function while avoiding membrane disrupting activity of peptide. In addition, unlike conventional antibiotics that target a specific biochemical process or a cell component, AMPs as a group have multiple modes of action, making them resilient against bacterial resistance [23,24].

Our current study provides an insight into the antimicrobial activity of DAN2 *in-vivo*. We found that DAN2 prolongs the survival of the mice in a dose dependent manner and more importantly, a single bolus dose protects infected mice from lethal bacterial challenge. The effective dose of DAN2 is comparable to other AMPs studied in the literature. A synthetic AMP named M33 (9 amino acid residues long) protected 100% of mice infected with lethal doses of *E*. *coli* and *P*. *aeruginosa* when administered at 12.5 and 25 mg/kg respectively [25]. Another study conducted in a rat model of septic shock demonstrated that a cecropin B reduced the lethality when given i. p. immediately after *E*.*coli* challenge at 1 mg/kg [26]. This suggests that the peptides belonging to the cecropin family can be effective *in vivo*. However, only a few *in vivo* studies of cecropins have been reported.

Several studies suggest that the cationic AMPs interact with the negatively charged membrane and form either ion channels or pores [23,24]. These cationic AMPs can also block intracellular processes by inhibiting protein folding or activity of enzymes, [14,27,28]. However, the cell membrane is reported as the primary target of cecropins [14]. We have found that DAN2 compromises the integrity of the bacterial membrane. Our *in vitro* study demonstrated that DAN2 does not lyse mammalian RBC which supports its potential use clinically [19]. Unlike bacterial cells which have 25% more anionic lipids that favor stronger electrostatic interaction, the mammalian cell membrane has large amounts of charge-neutral components, such as phosphatidylethanolamine, phosphatidylcholine, and sphingomyelin [29]. In addition, the presence of cholesterol in mammalian cell membranes stabilizes the phospholipid bilayer and hence reduces the pore-forming activity of AMPs.

While *in vitro* studies support lack of mammalian cell cytolysis by DAN2, it is possible that in vivo utilization causes toxicity through a mechanism other than cytolysis. To further evaluate potential toxicity of DAN2, we IP-injected C57BL/6 mice with 40 mg/kg (twice the highest concentration tested in the protection assay). The mice were observed over 5 days for behavioral changes associated with illness including weight loss, hunched posture, ruffled fur, and slow response to handling. However, there were no symptoms of illness (data not shown). After euthanasia the organs were observed by gross dissection and had no discernible abnormalities. Additionally, during treatment the infected mice that got sufficient DAN2 to protect them from the infection did not demonstrate murine illness behaviors and following euthanasia the organs appeared normal upon dissection. Our *in vivo* studies show that DAN2 is not toxic to mice when used to treat acute infection.

Several studies have suggested that AMPs work not only by disrupting the cell membrane, but also by exerting immune-modulatory activity[30–36]. To date, a class of cecropin family has been shown to stimulate the migration of leukocytes to the site of infection, reduce plasma levels of tumor necrosis factor, endotoxins, and cytokines responsible for septic shock [26,37–42] There has also been a report of increased anti-inflammatory cytokines (IL-4, IL-10) and/or reduced pro-inflammatory molecules (IL-6, IL-8, TNF-alpha) following an administration of cecropins [39,43]. These multiple modes of action of cecropin peptide awaits further investigation in the field.

As science has revealed the importance of normal flora in the host, the problem with disruption of normal flora from antibiotic use has been recognized. The standard antibiotics that are administered orally are disruptive of the normal flora [44]. However, there is considerably less information on the influence of AMPs on commensals. A report suggested that some commensal bacteria defend themselves against AMPs the host secretes by modifying the negatively charged phosphate group in their LPS [45]. Overall, there has been minimal investigation of the impact of IV or IP administration of AMP on the normal flora, and our study of intraperitoneal administration of DAN2 did not monitor normal flora changes. Our expectation is that while many commensals are likely susceptible to DAN2, IP administration would limit access of a charged peptide to the normal flora and impact on normal flora would be minimal.

An argument against the potential use of AMPs as anti-infective agent is the expected development of neutralizing antibodies when a given AMP is repeatedly applied to the same patient [46]. This is an important issue since antibodies against a given peptide would be likely to reduce the antimicrobial activity and since the peptide administration is temporally and perhaps physically associated with an infection, the immune system response against it is more likely to occur. One approach to avoid this problem is to not use a given peptide more than a few times in the same patient. The number of potentially clinically useful AMPs in nature is almost without limit. There could be hundreds of available peptides to treat any given infection and the repeated use of the same peptide could be minimized to avoid neutralizing antibodies.

To date, most of the characterized peptides have been identified in arthropods and vertebrates, particularly amphibian [47]. However, in virtually any organism in which they have been sought, novel AMPs have been found. Recently AMPs have also been identified in a broader range of organisms such as plants, *Feijoa sellowiana Berg* fruit [48] and shellfish *Mytilus galloprovincialis* mussel [49]. This argues that there is an inexhaustible supply of natural AMPs and we have barely begun to identify, catalog and test them experimentally and clinically. The developed technology to generate peptide in large quantities inexpensively further supports the potential for AMPs to contribute to new treatments for bacteria resistant to standard antibiotics. The availability of such a pharmacy of studied AMPs would provide a crucial tool to treat microbes that are resistant to standard small molecule antibiotics.

Our findings indicate that a cecropin type peptide, DAN2, has potential for clinical use. The present research is a further step in examining the antimicrobial activity of DAN2 in the process of developing this peptide as a therapeutic drug.

Funding information: Research was supported by New Hampshire-INBRE through an Institutional Development Award (IDeA), P20GM103506, from the National Institute of General Medical Sciences of the NIH.

## Supporting information

S1 Fig Survival percent of *E. coli* infected mice that received either 20 mg/kg of peptide or PBS after 30 minutes of bacterial challenge. All twelve mice were infected with a lethal dose of *E*. *coli* ATCC 25922 intraperitoneally (i.p.). The control group received 300μl of PBS after 30 minutes of bacterial challenge. Control mice showed 0% survivability, whereas 20 mg/kg peptide ensured 100% survivability in *E*. *coli* infected mice.
(TIFF)

S2 Fig Survival of C57BL/6 mice following *E. coli* infection and different doses of DAN2 over five days. The control group did not receive any peptide. Six mice were used per group. 5 mg/kg, 10 mg/kg and 20 mg/kg of peptide prolonged the survival of mice, but all control mice reached the endpoint within 12 hours of bacterial infections.
(TIFF)
