# Supplementary figures and images for: Cecropin-like antimicrobial peptide protects mice from lethal E.coli infection
Source: PLoS One. 2019 Jul 25;14(7):e0220344. doi: 10.1371/journal.pone.0220344 (PMC6658118; doi:10.1371/journal.pone.0220344)

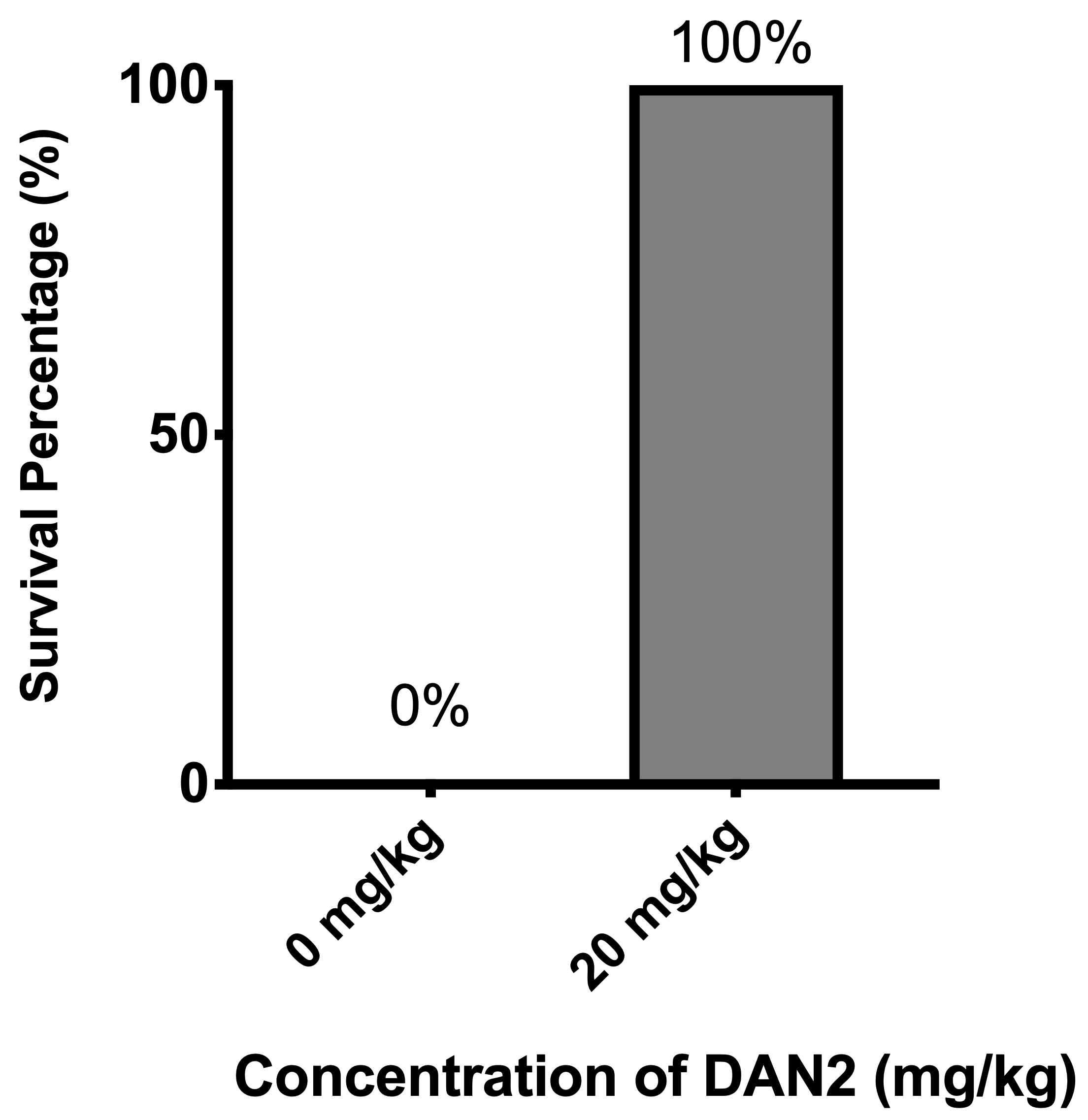

Supplement: S1 Fig — All twelve mice were infected with a lethal dose of E. coli ATCC 25922 intraperitoneally (i.p.). The control group received 300μl of PBS after 30 minutes of bacterial challenge. Control mice showed 0% survivability, whereas 20 mg/kg peptide ensured 100% survivability in E. coli infected mice. (TIFF) [file pone.0220344.s001.tiff]

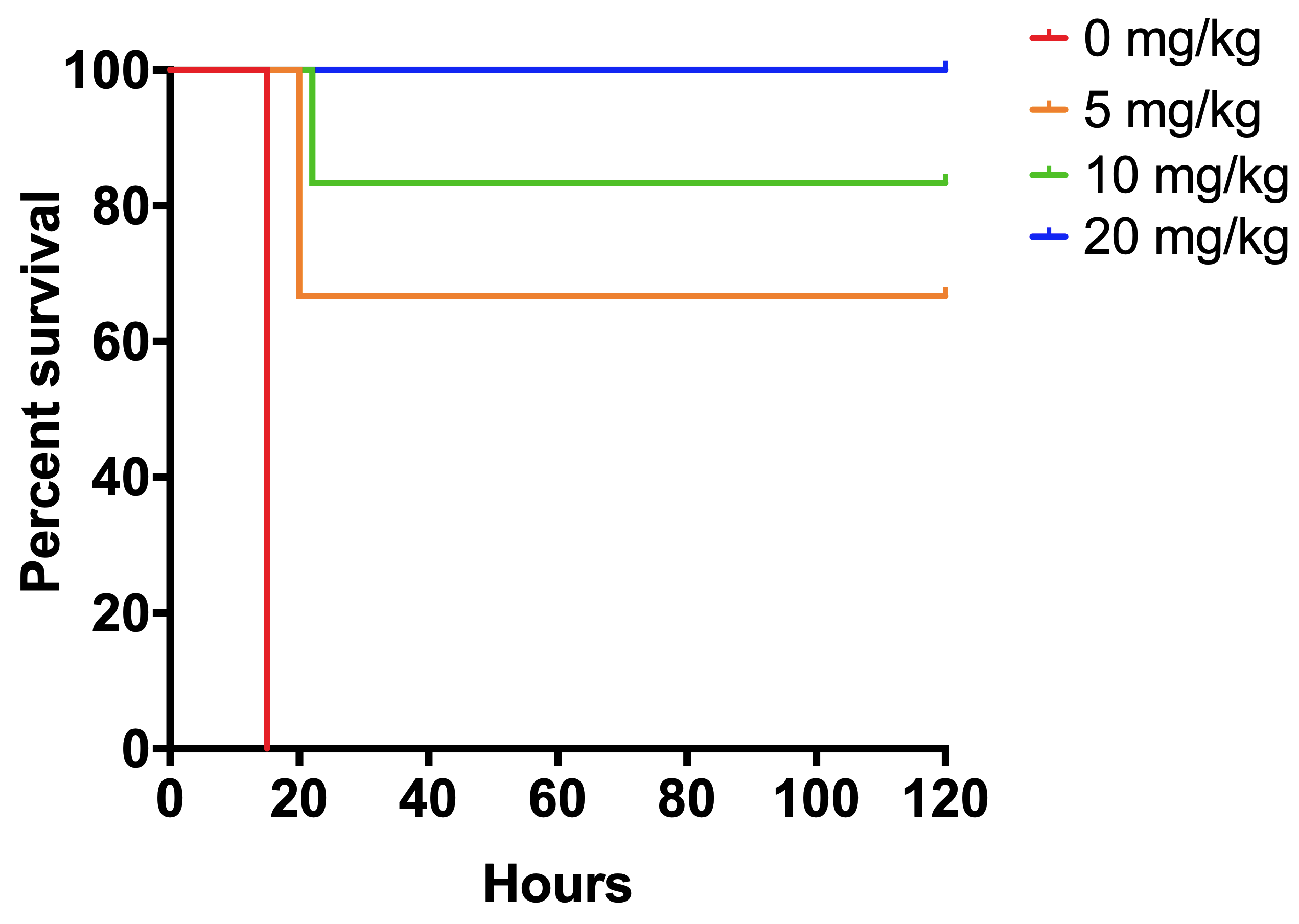

Supplement: S2 Fig — The control group did not receive any peptide. Six mice were used per group. 5 mg/kg, 10 mg/kg and 20 mg/kg of peptide prolonged the survival of mice, but all control mice reached the endpoint within 12 hours of bacterial infections. (TIFF) [file pone.0220344.s002.tiff]
